# Stable Isotope Tracing Analysis in Cancer Research: Advancements and Challenges in Identifying Dysregulated Cancer Metabolism and Treatment Strategies

**DOI:** 10.3390/metabo14060318

**Published:** 2024-05-31

**Authors:** Dalton Hilovsky, Joshua Hartsell, Jamey D. Young, Xiaojing Liu

**Affiliations:** 1 Department of Molecular and Structural Biochemistry, North Carolina State University, Raleigh, NC 27695, USA; dhilovs@ncsu.edu (D.H.); jchartse@ncsu.edu (J.H.); 2 Department of Chemical and Biomolecular Engineering, Vanderbilt University, Nashville, TN 37212, USA; 3 Department of Molecular Physiology and Biophysics, Vanderbilt University, Nashville, TN 37212, USA

**Keywords:** stable isotope tracing, high resolution mass spectrometry, cancer metabolism

## Abstract

Metabolic reprogramming is a hallmark of cancer, driving the development of therapies targeting cancer metabolism. Stable isotope tracing has emerged as a widely adopted tool for monitoring cancer metabolism both in vitro and in vivo. Advances in instrumentation and the development of new tracers, metabolite databases, and data analysis tools have expanded the scope of cancer metabolism studies across these scales. In this review, we explore the latest advancements in metabolic analysis, spanning from experimental design in stable isotope-labeling metabolomics to sophisticated data analysis techniques. We highlight successful applications in cancer research, particularly focusing on ongoing clinical trials utilizing stable isotope tracing to characterize disease progression, treatment responses, and potential mechanisms of resistance to anticancer therapies. Furthermore, we outline key challenges and discuss potential strategies to address them, aiming to enhance our understanding of the biochemical basis of cancer metabolism.

## 1. Introduction to Stable Isotope Tracing

Stable isotope tracing is a powerful technique used in metabolic research to investigate the pathways and dynamics of biochemical reactions within biological systems [1]. It involves labeling specific atoms within molecules, typically carbon, nitrogen, or hydrogen, with stable isotopes such as ^13^C, ^15^N, or ^2^H, respectively [2,3,4,5]. By tracking the fate of these labeled atoms through metabolic pathways, researchers can gain insights into various metabolic processes, including nutrient utilization, energy production, and biosynthesis. In the context of metabolic diseases, stable isotope tracing offers valuable insights into altered metabolic fluxes and aberrant pathways associated with conditions such as obesity, diabetes, and metabolic syndrome. By comparing the metabolism of labeled substrates in diseased versus healthy states, researchers can identify metabolic signatures, dysregulated pathways, and potential therapeutic targets. It is also useful in characterizing metabolic heterogeneity within the tumor microenvironment [6]. Overall, stable isotope tracing is an indispensable tool for unraveling the complexities of metabolic diseases, providing crucial information for understanding disease mechanisms and developing novel therapeutic interventions. Below, we outline the typical steps involved in conducting a stable isotope tracing assay.

### 1.1. Overview of Experimental Design of Stable Isotope Tracing Experiments

Unlike untargeted metabolomics analysis, which proceeds without the requirement of a hypothesis or a focus on a specific metabolic pathway, stable isotope studies begin with the design of a tracing protocol, which is optimized largely based on the metabolic pathways of interest. This process demands the careful consideration of factors such as tracer selection, labeling protocol, sampling time points, and tissue-specific metabolic dynamics.

Formulation of research question: Building upon prior discoveries and working with a formulated hypothesis often simplifies the process. However, for discovery studies lacking a pre-established hypothesis, it is essential to conduct untargeted metabolomics or other types of omics (e.g., gene expression) studies before performing a stable isotope tracing analysis [7,8]. These initial investigations help identify dysregulated metabolic pathways and formulate specific research questions that the stable isotope tracing experiment aims to address. This step is crucial for guiding the design of an appropriate isotope tracing study based on the knowledge of biochemical reactions involved in the pathway(s) of interest. Factors such as cell culture conditions, animal models, or human subjects should also be considered based on the specific research questions to be addressed.

Selection of tracer(s): The choice of appropriate stable isotopes (e.g., ^13^C, ^15^N, ^2^H, ^18^O) and specific labeled compounds for tracing the desired metabolic pathway requires an understanding of the metabolic reactions. The choice of tracers is highly dependent on the metabolic enzymes of interest. Stable isotope-labeled tracers that closely mimic endogenous metabolites provide accurate insights into metabolic fluxes. Many types of cancer cells utilize glucose and glutamine [9,10], and hence, when there is not a well-formulated hypothesis or a specific metabolic enzyme to monitor, uniformly labeled ^13^C tracers (e.g., [U-^13^C_6_]glucose or [U-^13^C_5_]glutamine) are frequently used to study central carbon metabolism. These tracers allow researchers to monitor the utilization of glucose or glutamine carbons for the biosynthesis of downstream metabolites [2,3]. While uniformly labeled tracers provide a broad view of metabolic flux, singly labeled tracers may be a good option for monitoring particular metabolic enzyme activities. For example, position-specific ^13^C tracers, such as [1-^13^C_1_]-pyruvate, result in unlabeled citrate via pyruvate dehydrogenase activity, while citrate labeled with a single ^13^C atom ([M+1] isotopologue) would indicate pyruvate carboxylase activity [11].

^15^N tracers are commonly used as stable isotope labels for tracking nitrogen metabolism, such as the incorporation of ^15^N into amino acids, nucleotides, or hexosamine. ^2^H tracers are primarily used for monitoring reactions involving isomerase reactions and dehydrogenase reactions with NADH or NADPH as co-factors [12,13]. ^2^H_2_O tracing is also widely used for quantifying gluconeogenesis and pathways leading to the de novo biosynthesis of macromolecules (e.g., fatty acids, proteins, and DNA), both in vivo and in vitro [14]. To track metabolism involving the use of oxygen or the production of reactive oxygen species, the use of ^18^O_2_ as the tracer can provide essential information [15,16]. Additionally, H_2_[^18^O] as a stable isotope carrier of ^18^O was used not only for monitoring phosphoryl turnover but also for tracking oxygen exchange in other reactions, such as the tricarboxylic acid (TCA) cycle [17]. It is important to consider the compatibility of tracers with analytical techniques. For example, using an instrument capable of distinguishing between ^13^C and ^15^N isotopologues is beneficial when performing ^13^C and ^15^N dual isotopic labeling experiments. A comprehensive review detailing the choice of stable isotope tracers and the corresponding metabolic reactions to be monitored is available elsewhere [18].

Labeling protocol optimization: To optimize isotopic enrichment while minimizing non-specific effects (e.g., insulin fluctuations) and metabolic perturbations, it is essential to tailor conditions such as tracer concentration, the route and timing of tracer administration, and labeling duration according to the metabolic activities and kinetics of the pathways of interest and the specific experimental models to be examined (cells, tumor slices, or whole organisms) [2,3,19]. In animal studies, tracer administration procedures should be assessed as certain techniques, such as anesthesia, have been demonstrated to impact specific metabolic activities [20]. It is also important to optimize the tracer dose to achieve sufficient measurement sensitivity while minimizing metabolic perturbations that can lead to experimental artifacts [21]. For monitoring metabolic activities with a fast turnover rate, such as glycolysis, a bolus injection or short-term infusion of ^13^C tracers is sufficient to achieve high enrichments in measured metabolites, but for monitoring protein or lipid synthesis, alternative tracer administration methods (e.g., via drinking water or diet) are necessary due to the slower turnover rate of these pathways [22]. Pilot experiments may be performed to capture metabolic dynamics under various conditions, guiding the definition of sampling time points based on pathway kinetics. Sample collection strategies should be designed to minimize metabolic disruption and post-harvest metabolite turnover, which is often achieved through snap-freezing in liquid nitrogen [23].

Sample processing and analysis: While errors should be managed throughout the process, analytical errors can typically be evaluated and minimized by integrating internal standards or incorporating quality control measures, such as the analysis of unlabeled samples or labeled standards to detect unexpected measurement interferences. In contrast, pre-analytical errors, such as delays in sample freezing or improper storage can compromise metabolite integrity and present significant challenges for monitoring and control. Strict adherence to sample collection and handling protocols is essential to minimize pre-analytical errors, preserve stable isotopic labeling, and maintain metabolite integrity.

Metabolites or lipids are typically extracted using organic solvents such as methanol–water mixtures, acetonitrile–water mixtures, or isopropanol–water mixtures, as well as more hydrophobic solvents like chloroform or methyl tert-butyl ether (MTBE) for lipids. Analytical techniques such as mass spectrometry (MS) and nuclear magnetic resonance spectroscopy (NMR) are commonly employed to measure isotopologues or isotopomers, respectively. Computational tools are used for calculating isotopic enrichment, correcting for natural isotopic abundance, or performing metabolic flux analysis to identify differences between experimental groups.

Validation and integration: The conclusions drawn from stable isotope tracing experiments require validation using complementary methods like genetic or pharmacological interventions. These interventions allow for the direct manipulation of key enzyme activities within dysregulated metabolic pathways, thus confirming their significant roles in cancer biology, such as influencing cancer proliferation or metastasis. Additionally, integrating stable isotope tracing data with other omics datasets, such as gene expression profiles or metabolite pool size measurements, facilitates a thorough comprehension of metabolic regulation and network interactions.

### 1.2. Analysis of Isotope Labeling with Nuclear Magnetic Resonance (NMR)

NMR spectroscopy: NMR spectroscopy is a powerful tool for analyzing stable isotope labeling patterns [24]. In NMR analysis, metabolites are exposed to radiofrequency pulses, and nuclei within the metabolites absorb energy and transition between various energy states. This process generates frequencies that offer insights into the chemical environment of the nuclei, facilitating the identification and quantification of metabolites. NMR-based isotope tracing has roots dating back to as early as the 1970s [25,26,27]. Isotopically labeled metabolites exhibit distinct NMR signals compared to their unlabeled counterparts due to changes in nuclear spin. By comparing the NMR spectra of labeled and unlabeled samples, researchers can identify isotopically enriched metabolites and quantify the extent of labeling, which allows for monitoring the fate of stable isotope-labeled tracers. For instance, in glucose metabolism studies, the incorporation of ^13^C-labeled glucose into downstream metabolites, such as lactate, citrate, glutamate, or alanine, can be monitored using NMR. Furthermore, unlike MS, NMR enables researchers to identify the positions of labeled atoms within molecules by analyzing the spectral patterns and chemical shifts of NMR peaks, providing insights into specific metabolic pathways, which is crucial for understanding pathways like glucose metabolism in which the position of the labeled atom may be indicative of its origin. By measuring the incorporation of labeled precursors into metabolites of interest, researchers can calculate flux rates and assess the activity of specific metabolic pathways.

Magnetic resonance imaging: Various non-invasive and nondestructive imaging techniques have been developed based on the principles of NMR [28,29,30,31]. Traditional magnetic resonance imaging (MRI) essentially measures hydrogen atoms, which are abundant in biological organisms, especially in water and fat, and provides information about the shape and internal structure of soft tissues [32,33]. Due to sensitivity issues, in many instances, MRI cannot directly monitor other metabolites in tissues unless they are isolated and enriched. The spatial resolution of metabolism has been achieved by combining PET-CT scanning or MRI with metabolite extraction from dissected tumors, followed by NMR or MS-based analysis [34]. By introducing hyperpolarized ^13^C tracers, hyperpolarized MRI (HP-MRI) has improved sensitivity [35] and enables the non-radioactive and non-invasive monitoring of metabolic processes in vivo, offering real-time metabolic information with spatial resolution [28,29,30,31,36]. HP-MRI provides insights into regional metabolic heterogeneity and facilitates the characterization of metabolic phenotypes associated with diseases such as cancer. For instance, MRI has advanced the understanding of glutamine metabolism in tumor xenograft models, highlighting the heterogeneity of tumor metabolism [37,38]. It has been used to noninvasively monitor pyruvate metabolism in prostate and breast cancers in the clinical setting [39,40,41].

Advantages: NMR-based metabolomics is nondestructive, allowing for the repeated analysis of the same sample without significant sample loss [42]. In contrast, MS-based methods involve ionization and fragmentation, altering the sample irreversibly with each analysis. Additionally, since the intensity of an NMR signal correlates directly with the number of nuclei responsible for a particular resonance, NMR inherently yields quantitative data [43,44]. An underappreciated aspect of NMR is its ability to accurately quantify low-isotope enrichments (e.g., ~0.1%) that are below the noise threshold of typical MS measurements [45]. Furthermore, NMR’s instrument-independent chemical shifts contribute to greater reproducibility of metabolomics results across different laboratories, allowing for data exchange among instruments, especially those with comparable hardware, or even data from different laboratories and with quite variable skills [44,46].

Limitations: NMR-based metabolomics does have limitations. NMR typically exhibits lower sensitivity compared to MS-based methods, resulting in fewer identified metabolites [42,47,48]. At the moment, the availability of NMR-specific data processing software may be more limited compared to MS-based metabolomics [42,49,50]. Despite these drawbacks, NMR remains a valuable tool for metabolomic studies, particularly for its nondestructive nature, reproducibility, and capability for spatial metabolite imaging.

### 1.3. Analysis of Stable Isotope Labeling Using Mass Spectrometry (MS)

Mass spectrometry: In addition to NMR-based metabolomics, MS, especially high-resolution mass spectrometry (HRMS), is a powerful analytical technique widely employed for studying stable isotope labeling in metabolomics research [2,5,51]. MS measures the mass-to-charge ratio (*m*/*z*) of ions generated from molecules through ionization techniques such as electrospray ionization (ESI). Isotopically labeled metabolites exhibit distinct mass shifts compared to their unlabeled counterparts due to the incorporation of heavier isotopes, such as ^13^C, ^15^N, ^2^H, or ^18^O. Well-calibrated HRMS instruments with high mass accuracy and resolution ensure the reliable detection and quantification of isotopically enriched metabolites, even at low abundance levels. Most MS-based metabolomics approaches typically employ liquid chromatography mass spectrometry (LC-MS) coupled with electrospray ionization (ESI). However, alternative separation methods, such as gas chromatography (GC), have also been used [52,53,54], offering complementary capabilities for analyzing certain metabolites, such as volatile metabolites. Additionally, tandem mass spectrometry (MS/MS) is commonly employed in MS-based metabolomics to facilitate metabolite identification. It involves the fragmentation of precursor ions to provide structural information, which can offer insights into the position of stable isotopes within the metabolite molecules [55]. Comprehensive reviews of LC-MS metabolomics method development and recent advancements in the field have been previously documented [56,57,58].

Mass spectrometry imaging: Even though MS data typically lack spatial resolution, efforts to address this limitation have led to the development of mass spectrometry imaging (MSI) [59,60,61]. MSI typically utilizes matrix-assisted laser desorption ionization (MALDI) [60] or related techniques, such as desorption ionization electrospray (DESI) [61]. MSI has proven valuable in measuring metabolomic flux in both healthy and tumorous tissues, revealing spatial heterogeneity in metabolic phenotypes [62,63]. However, compared to MRI, MSI-based techniques typically require the isolation of tissue sections [62,63] and have not yet been widely applied for the direct measurement of in vivo flux, even though efforts are made to advance the in vivo applications [64,65,66].

Advantages: The major advantage of MS is that MS methods offer high sensitivity and resolution compared to NMR, enabling the detection and identification of thousands of metabolites from a complicated biological sample [42,67]. In addition, the MS can be coupled with a variety of separation platforms, such as LC, GC, ion chromatography (IC), supercritical fluid chromatography (SFC), and capillary electrophoresis (CE), which help to resolve individual chemical components and improve sensitivity. 

Limitations: However, there are several weaknesses to MS approaches. In contrast to NMR, MS-based metabolomics typically tracks isotopologues, and MS1 scans cannot resolve the positional isotopic isomers of a given metabolite because MS detects the mass-to-charge ratio rather than the molecular structure. In contrast, NMR spectroscopy can provide information about the spatial arrangement of atoms within a molecule, allowing for the resolution of positional isotopic isomers. Additionally, MS itself is not inherently quantitative. The intensity of MS signals can be influenced by various experimental factors, such as ionization efficiency, matrix effects, and instrument settings, and hence, directly comparing datasets from different batches or across laboratories can be challenging [68,69,70]. However, it is worth noting that Clark et al. report qualitatively similar PCA plots in their interlaboratory study, indicating that while direct data comparison is often difficult, the overall trends observed in data across laboratories largely align [69]. Additionally, the impact of interlaboratory variables on the MS signal readout of isotopologues or isotopic isomers of the same metabolite is consistent, and hence, variations are minimized when labeling percentages, rather than relative intensities, are reported in the stable isotope-tracing analysis. 

Because of these complementary capabilities of MS and NMR, efforts have been made to enable their coupling in metabolomics analysis with enhanced confidence in metabolite identification [49,71]. Publicly available software for modeling combined MS and NMR datasets has been developed, which provides improved precision when applied to estimate metabolic fluxes from stable isotope tracing experiments [72]. 

### 1.4. Data Analysis

NMR or MS raw data analysis: Once NMR- or MS-based metabolomic data are collected, they undergo several preprocessing steps before statistical analysis. The preprocessing steps include baseline correction and solvent peak suppression to minimize experimental variability, followed by alignment of spectral peaks. In MS data, alignment occurs along the retention time of known features, whereas in NMR spectra, alignment is based on a chemical shift. Depending on the nature of the data, normalization may also be necessary, which can be achieved through various methods, such as using an internal standard or total ion chromatogram for normalization. Metabolite identification in stable isotope tracing studies follows a similar process to general metabolomics. For example, stable isotope labeled metabolite identification also relies on features like mass-to-charge ratio (*m*/*z*), MS/MS fragmentation patterns, and chromatographic retention time. Identification based on the isotope pattern may not be applicable in stable isotope tracing experiments because of the incorporation of exogenous stable isotopes. Therefore, it is important to analyze unlabeled control samples alongside labeled samples to facilitate metabolite identification based on comparisons to spectral libraries. In-depth discussions of spectral alignment and metabolite identification steps have already been covered elsewhere [44,67,73,74]. In addition to data processing software provided by vendors of NMR or MS instruments, several open-source software options are available for metabolomic data analysis. For NMR data, tools like rDolphin and AQuA are used for targeted or semi-targeted metabolite analysis [75,76], while tools like AlpsNMR or SigMa offer functionalities for processing and analyzing NMR-based untargeted metabolomics data [77,78]. Open-source tools, such as MAVEN, Skyline, and PIRAMID, or vendor-specific commercially available software (e.g., TraceFinder, MassHunter, etc.) are widely used to analyze data collected using targeted MS acquisition methods such as multiple reaction monitoring (MRM) or the extraction of targeted metabolite information from data collected using an untargeted HRMS method [79,80,81]. Using these tools for targeted metabolite analysis usually requires a file containing targeted metabolite mass-to-charge ratio, metabolite retention times, or MRM scan parameters to retrieve peaks from raw data files. For MS-based untargeted metabolomics data analysis, open-source platforms like XCMS, MetaboAnalyst, MS-DIAL, and MZmine provide comprehensive solutions for the peak detection, alignment, normalization, and annotation of MS data [73,82,83,84]. More specifically for stable isotope tracing data analysis, software tools such as X13CMS, DynaMet, geoRge, HiResTEC, MetTracer, and others offer a global analysis of ^13^C enrichment in metabolites [85,86,87,88,89]. X13CMS is an extension of XCMS, leveraging its output to identify isotopologue groups corresponding to isotopically labeled compounds [85]. Beyond ^13^C, X13CMS can analyze other isotope-labeled metabolites, making it a versatile tool for stable isotope tracing studies. A comprehensive review of software options for metabolomics data analysis can be found elsewhere [50].

Natural abundance correction: Since many stable isotopes used for labeling studies are naturally occurring at measurable levels, the mass isotopologue distribution (MID) measured by MS does not directly reflect the isotope enrichment from exogenous isotope tracers, and correcting for naturally occurring isotopic abundances is important when analyzing labeling data. This correction ensures that the observed isotopic distributions are attributed solely to the introduced tracer, allowing for the precise quantification and interpretation of metabolic fluxes and pathways. Contemporary methods for natural abundance correction integrate the observed MID with the theoretical isotope abundance of the analyte’s constituent atoms to solve for the corrected MID using a linear transformation or the least-squares regression method. Since natural abundance corrections can be computationally intensive, several software tools have been developed to facilitate this process [90,91,92]. PolyMID-Correct and AccuCor2 are open-source tools that can be used to handle data with dual-isotope tracers and data collected on low- and high-mass-resolution mass spectrometers. IsoCorrectoR is primarily used for the natural abundance correction of multiple-tracer data, such as ^13^C and ^15^N, collected using HRMS at the MS1 and/or MS2 level [93], and IsoCor v2 is designed for data with any resolution [91]. It is important to note that some tools, like IsoCorrectoR, assume that all non-tracer isotopologues are resolved from tracer isotopologues. However, depending on the mass of the analyte and the resolving power of the instrument used, some non-tracer isotopologues may not be resolved from tracer isotopologues, even with a high-resolution instrument. For example, if ^13^C_2_ is incorporated into acetyl-CoA, the mass difference from [^18^O_1_]-acetyl-CoA arising from natural abundance of oxygen is only 0.00241, and given that the *m*/*z* of [^13^C_2_]-acetyl-CoA is 812.13976 in positive mode, a resolving power of at least 336,929 would be required to distinguish [^13^C_2_]-acetyl-CoA from [^18^O_1_]-acetyl-CoA. An in-depth discussion of the mathematical calculations involved in natural abundance correction and the potential impact of corrected MIDs on metabolic flux analysis is elegantly provided elsewhere [90,94]. 

Data visualization and mathematical modeling: In some cases, a direct interpretation of the data, such as simply plotting the abundance of measured isotopomers or isotopologues, is sufficient to provide insights into metabolic alterations, especially for simpler systems or when focusing on specific metabolites. These plots can be made manually, or a web-based tool can be employed for a pathway-based visualization of stable isotope tracing data [95]. Nevertheless, the modeling of stable isotope tracing offers a more comprehensive and quantitative approach [96,97,98,99,100]. By integrating metabolite measurements with computational models that incorporate mass conservation constraints, researchers can gain a deeper understanding of metabolic network dynamics and regulation. Flux balance analysis (FBA) and metabolic flux analysis (MFA) are two commonly used computational approaches in systems biology for predicting and analyzing metabolic fluxes in biological systems. FBA assumes that cellular metabolism operates at steady state, with the rates of metabolite production and consumption balanced, and FBA predicts fluxes based on biochemical and physiological knowledge, such as reaction stoichiometry, thermodynamics, enzyme capacity constraints, and optimality assumptions [101]. On the other hand, MFA estimates metabolic reaction rates and flux distributions by fitting mathematical models to experimental data obtained from stable isotope labeling experiments [98]. Commonly used software tools for ^13^C MFA analysis include 13CFLUX2, FiatFlux, Metran, INCA, and others [102,103,104,105,106,107,108,109]. A detailed review of MFA analysis and commonly used software tools can be found in previous publications [110,111]. 

Interpretation and hypothesis generation: The results obtained from fluxomics analysis can be used to test existing hypotheses or generate new ones about how metabolic pathways are regulated and their overall function within the system under study [1,98]. However, to validate these hypotheses and further refine our understanding of metabolism, experimental testing is essential. Experimental validation could involve a variety of approaches. For example, genetic or pharmacological manipulations using gene knockout, knockdown, or overexpression, as well as the use of small molecule inhibitors or activators, can be employed to directly manipulate the enzymes involved in metabolic pathways. By perturbing the expression or activity of these enzymes, researchers can assess the impact on metabolic fluxes and validate hypotheses regarding the importance of these enzymes in pathway regulation. Additionally, measuring cancer cell physiology, such as proliferation rate, metastasis potential, and response to anticancer treatments, is also necessary to validate the regulatory role of a particular metabolic enzyme or pathway in cancer biology.

## 2. The Applications of Stable Isotope Tracing in Cancer Research

The use of stable isotope tracing in cancer metabolism studies is invaluable for identifying potential drug targets that hinder metabolic reprogramming, including the upregulation of glycolysis, the pentose phosphate pathway (PPP), the TCA cycle, and nucleotide biosynthesis. In this section, we mainly focus on reviewing the applications of stable isotope tracing using ^13^C glutamine, ^15^N glutamine, and ^13^C glucose for understanding cancer metabolism. Due to space limitations, we primarily discuss studies published within the last three years, and we apologize for any studies not cited here. In vitro or ex vivo, isotope labeling usually involves the straightforward substitution of unlabeled nutrients with their labeled counterparts (e.g., replacing glucose with ^13^C-labeled glucose) [19,112], while the method for in vivo isotope tracing varies depending on the tracer and the metabolic pathways of interest [3,113,114]. 

### 2.1. Exploring Cancer Metabolism through Glutamine Isotope Tracing

**Figure 1:**
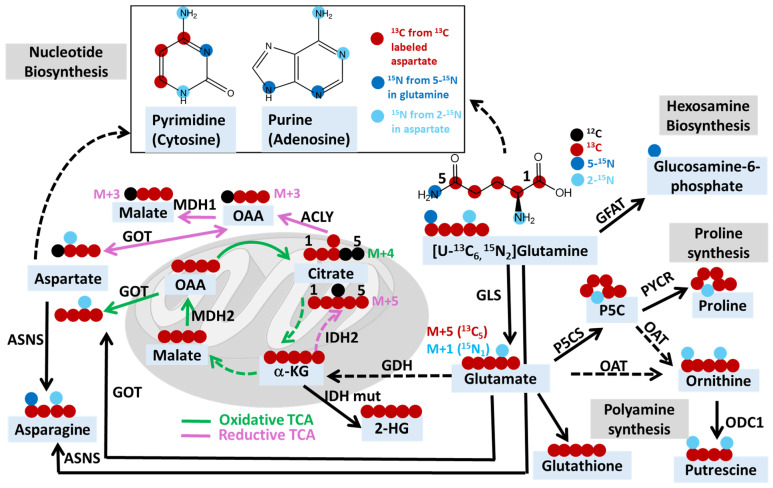
Schematic depicting the potential fates of ^13^C and ^15^N in [U-^13^C_6_, ^15^N_2_]glutamine. Abbreviations: MDH, malate dehydrogenase; ACYL, ATP citrate lyase; IDH, isocitrate dehydrogenase; IDH mut, IDH mutant; GDH, glutamate dehydrogenase; GLS, glutaminase; GFAT, fructose-6-phosphate amidotransferase; P5CS, pyrroline-5-carboxylate synthase; PYCR, Δ1-pyrroline-5-carboxylate reductase; OAT, ornithine aminotransferase; ODC1, ornithine decarboxylase 1; GOT, glutamate-oxaloacetate transaminase 2; ASNS, asparagine synthetase; OAA, oxaloacetate; α-kG, alpha-ketoglutarate; 2-HG, 2-hydroxyglutarate; P5C, pyrroline-5-carboxylate.

Stable isotope tracing utilizing ^13^C- or ^15^N-labeled glutamine, coupled with various analytical techniques, such as LC-MS, GC-MS, or NMR, has been widely used for quantitatively monitoring glutamine metabolism in cancer research (Figure 1). Glutamine plays a pivotal role in the survival and proliferation of cancer cells by serving as a crucial carbon and nitrogen source [9,115]. This multifaceted role contributes to various metabolic pathways essential for tumor growth. Glutamine is utilized in cancer cells to provide carbon for the synthesis of lipids and metabolites in the TCA cycle, as well as nitrogen for the biosynthesis of hexosamine, amino acids, and nucleotides, thus supporting the demands of cancer cell proliferation and growth [115,116,117]. Glutamine is converted to glutamate through glutaminolysis catalyzed by glutaminase (GLS). Subsequently, glutamate is further deaminated to α-ketoglutarate (αKG) through the action of enzymes such as glutamate dehydrogenase (GDH) or various transaminases, including glutamate-oxaloacetate transaminase (GOT), glutamate-pyruvate transaminase (GPT), and phosphoserine transaminase (PSAT) [9,115]. Once present in the TCA cycle, αKG can be oxidized to generate four-carbon units or undergo reductive carboxylation to isocitrate and citrate, eventually leading to the synthesis of fatty acids [115,117]. 

By using [U-^13^C_5_]-glutamine, researchers can track the flux of carbons from glutaminolysis via GLS to oxidative and reductive TCA metabolites (Figure 1). Specifically, they can analyze the isotopic labeling patterns of metabolites, such as glutamate (M+5) and citrate (M+4) from oxidative TCA cycle flux and citrate (M+5) from reductive carboxylation flux, to determine the extent of glutamine-derived carbon incorporation [118]. [1-^13^C]glutamine can be applied to specifically confirm reductive carboxylation activity since glutamine C1 is lost as CO_2_ in the oxidative metabolism of αKG. However, it is important to measure isotope incorporation in sink products such as palmitate or 2-hydroxyglutarate (2-HG) and apply comprehensive flux modeling to determine the direction and magnitude of the isocitrate dehydrogenase (IDH) flux since some labeling in citrate results from reversible exchange with αKG rather than net reductive flux [119]. Glutamine contains the following two nitrogens: 5-N or amide-N and 2-N or amine-N. Glutamine with ^15^N labeling at the five position ([5-^15^N]glutamine, or [Amide-^15^N]glutamine) can be used to monitor nitrogen incorporation into amino acids, such as asparagine, nucleotides, and hexosamine, while ([2-^15^N]glutamine, or [Amine-^15^N]glutamine leads to the ^15^N labeling at the two position of amino acids, such as alanine, aspartate, etc. 2-^15^N labeling in aspartate is subsequently incorporated into newly synthesized nucleotides [8,120]. Uniformly ^15^N-labeled glutamine ([U-^15^N_2_]glutamine) offers a comprehensive ^15^N labeling strategy to simultaneously enrich a variety of downstream nitrogen-containing metabolites. 

Metabolic flexibility: Stable isotope tracing with ^13^C- or ^15^N-labeled glutamine helps to understand the metabolic flexibility of cancer cells and provides insights into combination therapies that target multiple metabolic vulnerabilities simultaneously, thereby improving the effectiveness of antitumor treatments [121]. In one study, the use of a [U-^13^C_5_]-glutamine tracing analysis of osteosarcoma cells enabled researchers to investigate the contribution of the reductive carboxylation of glutamine-derived α-ketoglutarate to aspartate production, which helped to explain why osteosarcoma cells with deficiencies in succinate dehydrogenase (SDH), also known as electron transport chain (ETC) complex 2, continue to produce and rely on aspartate synthesis for proliferation [122]. When [U-^13^C_5_]-glutamine was used as the tracer, the knockout (KO) of SDH decreased the oxidative production of aspartate (M+4) but showed an increase in aspartate (M+3) derived from reductive carboxylation of glutamine-derived α-ketoglutarate in osteosarcoma cells [122]. The inhibition of ETC complex 1 increased reductive carboxylation and pyruvate carboxylation-derived aspartate production and rescued cell proliferation in osteosarcoma cells with SDS deficiency [122]. In another study, SDH-deficient cells have been shown to rely on glutamate–pyruvate transaminase (GPT2) for proliferation through GPT2-dependent α-ketoglutarate and alanine production [123], which was monitored through isotope tracing using [U-^13^C_5_]glutamine and [2-^15^N]glutamine. GPT2 drives reductive TCA anaplerosis and regenerates NAD+ to support glycolysis [123]. [U-^13^C_5_]glutamine tracing confirmed that the inhibition of GPT2 in SDH-deficient cells decreased the levels of citrate (M+5) and cytosolic malate (M+3), accompanied by halting cancer proliferation [123]. In another study, an unbiased screening approach was employed to elucidate the adaptive mechanisms of cancers in response to glutamine deprivation, and this approach facilitated the identification of ALDH18A1, which encodes P5CS, the rate-limiting enzyme in the proline biosynthetic pathway, as a gene that cells can downregulate in response to glutamine starvation [124]. The subsequent [U-^13^C_5_]glutamine and [U-^13^C_6_]glucose tracing analysis confirmed decreased proline synthesis, which enabled carbon to be redirected toward de novo glutamate and glutamine synthesis, supporting the proliferation of glutamine-restricted cancer cells [124]. 

Metabolic heterogeneity: It is acknowledged that tumors display significant levels of genomic and metabolic heterogeneity [125,126,127]. Stable isotope tracing is of great value in examining both inter- and intratumoral metabolic heterogeneity. For example, stable isotope tracing assays with [U-^13^C_5_]glutamine in precision-cut slices of patient-derived xenografts (PDX), followed by GC-MS analysis, demonstrated the metabolic heterogeneity of human prostate tumors [128]. In another study, multi-isotope imaging mass spectrometry with isotope ratio mass spectrometry was used to analyze tumors isolated from mice bearing melanoma and malignant peripheral nerve sheath tumors injected with multiple tracers, including ^15^N-labeled glutamine [129]. The ^15^N labeling was monitored through an increase in the ^12^C^15^N^−^/^12^C^14^N^−^ ratio [129]. The results revealed tumor metabolic heterogeneity [129]. 

Metabolic requirements in tumor metastasis: CB-839 is a GLS inhibitor and reduces the conversion of glutamine to glutamate [130]. CB-839, when combined with metformin, results in both primary osteosarcoma growth inhibition and a notable reduction in metastatic outgrowth in vivo [131]. A [U-^13^C_6_,^15^N_2_]glutamine tracing analysis confirmed that the diminished M+4 or M+2 TCA intermediates without affecting M+5 or M+3 TCA metabolites, indicating a reduction in oxidative TCA cycle activity but no effect on reductive activity [131]. In another study, a gene expression analysis of breast cancer (BT474) growth in various tissues suggested the upregulation of genes related to lipid metabolism in breast tumors growing in the brain [7]. A subsequent [U-^13^C_6_]glucose tracing analysis with GC-MS and MALDI-MSI confirmed the elevated fatty acid synthesis. The genetic and pharmacological inhibition of FASN suppresses breast cancer growth in the brain, indicating that fatty acid synthesis could potentially serve as a therapeutic target for breast cancer brain metastases [7].

Tumor microenvironment: Stable isotope tracing using [U-^13^C_5_]glutamine in vitro and in vivo has been employed to investigate the interplay between tumor metabolism and immunogenicity within the tumor microenvironment (TME) and the association with immunogenicity [132]. The findings indicate distinct metabolic profiles between immunologically “hot” and “cold” melanoma tumors, with immunologically hot tumors exhibiting increased utilization of glutamine compared to immunologically cold tumors, both in vivo and in vitro [132]. The results not only highlight the potential significance of glutamine metabolism activity as a prognostic factor in melanoma but also offer insights for designing metabolic therapies to enhance immunotherapy efficacy [132]. In another study, a [U-^13^C_5_]glutamine tracing analysis revealed that proline originates from glutamine metabolism in cancer-associated fibroblasts, supporting the production of pro-tumorigenic collagen [133]. Decreasing proline synthesis by reducing the level of pyrroline-5-carboxylate reductase 1 (PYCR1) in cancer-associated fibroblasts is sufficient to reduce tumor collagen production, tumor growth, and metastatic spread in vivo [133].

Drug target engagement: Advancements in understanding glutamine metabolism have paved the way for identifying potential drug targets aimed at disrupting cancer proliferation by targeting enzymes involved in glutaminolysis [9,115,134]. As of now, the primary drug targets for inhibiting the incorporation of glutamine carbons into the TCA cycle and nitrogens into nucleotide and amino acid synthesis are glutaminase (GLS) and amidotransferases. Researchers have monitored the efficacy of inhibiting various enzymes associated with the TCA cycle, glutaminolysis, amino acid synthesis, and nucleotide synthesis using isotopes of glutamine, such as [U-^13^C_5_] and [5-^15^N]glutamine (Table 1) [98,118,131,135,136,137].

The effect of CB-839 on glutamine metabolism was confirmed by using [U-^13^C_5_]glutamine or [U-^13^C_5_,^15^N_2_]glutamine in sarcoma cells [131,138], ovarian cancer cells [139], prostate cancer cells [140], glioblastoma cells [136], and patient-derived xenografts from renal cell carcinoma [118]. Additionally, HP-MRI using [5-^13^C,4,4-^2^H_2_,5-^15^N]-L-glutamine was employed for monitoring glutamine metabolism in pancreatic cancer xenograft models and confirmed the reduced glutamine conversion to glutamate in vivo by CBP-839 [37]. 

6-Diazo-5-oxo-l-norleucine (DON), recognized as a glutamine antagonist, along with its prodrug, DRP-104, effectively inhibits the glutamine-dependent metabolism [141,142,143,144]. The impacts of DRP-104 on TCA cycle intermediates or nucleotide biosynthesis were verified through a [U-^13^C_5_]glutamine or [U-^13^C_5_,^15^N_2_]glutamine flux analysis in mice bearing lymphoma EL4 tumors [143], a syngeneic model of pancreatic ductal adenocarcinoma (PDAC) [143], or Keap1 mutant lung cancer cells [145]. Specifically, the decrease in the enrichment of the M+4 TCA intermediates (e.g., succinate, fumarate, and malate) in DRP-104–treated tumors compared to the control indicates that DRP-104 impeded the influx of glutamine-derived glutamate into the TCA cycle [143].

Dihydroorotate dehydrogenase (DHODH), an enzyme involved in the de novo pyrimidine nucleotide synthesis pathway, has been specifically targeted in cancer cells with IDH mutations. An analysis of [5-^15^N]glutamine metabolism has revealed that the inhibition of DHODH effectively halts nucleotide synthesis, particularly of pyrimidine nucleotides, thereby impeding cancer cell proliferation [146]. 

**Table 1:** Representative examples of isotope tracing analysis in characterizing cancer responses to treatments targeting metabolic enzymes.

Drug Target	Isotope Tracer	Pathway	Key Metabolite(s)	Cancer/Cell Type	Reference
Glutaminase (GLS)	[U-^13^C_5_]glutamine [U-^13^C_5_, ^15^N_2_]glutamine	Oxidative TCA Cycle	alpha-ketoglutarate (M+5), other TCA metabolites (M+4)	Renal cell carcinoma tumorgrafts	[118]
		Reductive TCA Cycle	alpha-ketoglutarate (M+5), citrate (M+5), other TCA metabolites (M+3)		
	[5-^15^N]glutamine	Amidotransferase	Asparagine (M+1)		
Glutamine-using enzymes	[U-^13^C_5_]glutamine	Glutamine contribution to TCA cycle	alpha-ketoglutarate (M+5), other TCA metabolites (M+4)	Lymphoma xenograft	[143]
Ornithine aminotransferase	[2-^15^N]glutamine	Ornithine and polyamine synthesis	Ornithine and putrescine (M+1 and M+2)	Xenografts of pancreatic ductal adenocarcinoma	[147]
Glutamine-fructose-6-phosphate transaminase 2	[5-^15^N]glutamine	Hexosamine biosynthesis	GlcNAc-6-P, UDP-HexNAc and ManNAc	KRAS/LKB1 co-mutant lung cancer cells	[8]
PYCR1, P5CS	[U-^13^C_5_]glutamine	Proline biosynthesis	Proline (M+5)	Cancer-associated fibroblasts, gastric cancer cell	[124,133]
IDH1 Mutations	HP [1-^13^C]glutamine	2-Hydroxyglutarate (2-HG) formation	Ratio of [1-^13^C] 2-HG to ^13^C urea	Chondrosarcoma xenograft with IDH1 mutation	[38]
Dihydroorotate dehydrogenase	[5-^15^N]glutamine	De novo pyrimidine synthesis	Uridine monophosphate (M+1)	IDH mutant gliomas	[146]
Alanine transaminase	[U-^13^C_6_]isoleucine, [U-^13^C_6_]leucine, [U-^13^C_5_]valine	BCAA contribution to TCA cycle	TCA metabolites (M+2 and M+3)	Melanoma-bearing zebrafish	[148]
ETC Complex 1	[U-^13^C_5_]glutamine	Reductive TCA cycle	alpha-ketoglutarate (M+5), citrate (M+5), other TCA metabolites (M+3)	Neuroblastoma xenograft	[149]
		Oxidative TCA cycle	alpha-ketoglutarate (M+5), other TCA metabolites (M+4)		
Autophagy Related 5 (ATG5)	[U-^13^C_3_]lactate	Gluconeogenesis	Glycolytic intermediates (M+3 or M+6)	KP lung tumor bearing mice	[150]
		Serine biosynthesis	Serine (M+3)		
Fatty acid synthase (FASN)	[U-^13^C_6_]glucose	De novo lipogenesis	Palmitate (M+n) n = 2, 4, 6, etc.	Mice carrying breast cancer with brain metastases	[7]

### 2.2. The Use of Other Isotope Tracers for Investigating Cancer Metabolism

**Figure 2:**
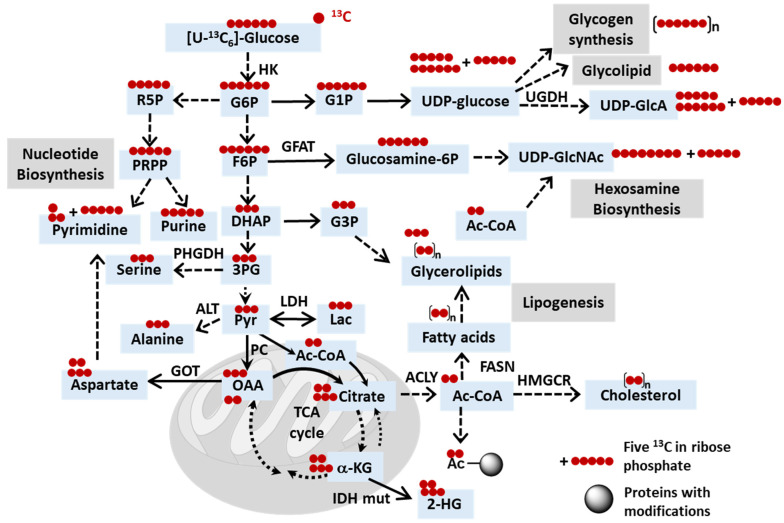
Schematic depicting the potential fates of ^13^C in [U-^13^C_6_]glucose. Abbreviations: HK, hexose kinase; ACYL, ATP citrate lyase; IDH, isocitrate dehydrogenase; IDH mut, IDH mutant; UGDH, UDP-glucose dehydrogenase; GFAT, fructose-6-phosphate amidotransferase; PHGDH, phosphoglycerate dehydrogenase; LDH, lactate dehydrogenase; ALT, alanine transaminase; FASN, fatty acid synthase; GOT, glutamate-oxaloacetate transaminase 2; HMGCR, 3-hydroxy-3-methylglutaryl-CoA reductase; Ac, acetyl; G6P, glucose-6-phosphate; G1P, glucose-1-phosphate; F6P, fructose-6-phosphate; R5P, ribose-5-phosphate; PRPP, phosphoribosyl pyrophosphate; UDP-GlcA, UDP-glucuronate; UDP-GlcNAc, UDP-N-acetylglucosamine; DHAP, dihydroxyacetone phosphate; G3P, glycerol-3-phosphate; 3PG, 3-phosphoglycerate; Pyr, pyruvate; Lac, lactate; OAA, oxaloacetate; a-kG, alpha-ketoglutarate; 2-HG, 2-hydroxyglutarate.

Glucose tracers: The Warburg effect is the hallmark of cancer and is characterized by the preferential utilization of glycolysis over oxidative phosphorylation, even in the presence of oxygen [151]. The significance of glucose in supporting the survival of proliferating cancer cells underscores the critical role of glucose isotope tracers, such as [U-^13^C_6_]glucose, in elucidating dysregulated cancer metabolism and monitoring metabolic responses to anticancer treatment (Figure 2). By studying the incorporation of carbon from [U-^13^C_6_]glucose into glycolysis, the TCA cycle, the PPP, amino acids such as serine, or newly synthesized fatty acids, researchers can gain valuable insights into metabolic dynamics (Figure 2 and Table 1). Similar to what has been discussed in Section 2.1, [U-^13^C_6_]glucose tracing serves not only to elucidate tumor metabolism but also to explore the interplay with the surrounding tumor environment and cells. For example, cancer cells harboring mutant IDH are known to overproduce D-2-hydroxyglutarate (D-2HG), which has been shown, through the isotope-tracing of [U-^13^C_6_]glucose and [U-^13^C_3_]lactate, to modulate glycolysis in CD8+ T cells by inhibiting lactate dehydrogenase (LDH) [152]. In another study, [U-^13^C_6_]glucose together with N-[1,2-^13^C_2_]acetyl-d-glucosamine revealed that in a PDA tumor microenvironment with limited glutamine and glucose, the hexosamine salvage played an important role in supporting hexosamine biosynthesis and PDA tumor proliferation [153]. 

M+n denotes the incorporation of n ^13^C- or ^15^N-heavy isotopes. For example, M+4 citrate indicates the citrate containing four ^13^C and two ^12^C. For metabolites containing both ^13^C and ^15^N, such as [^13^C_5_, ^15^N_1_]glutamate derived from [U-^13^C_5_, ^15^N_2_]glutamine, M+5 glutamate indicates glutamate with five ^13^C, while M+1 glutamate indicates glutamate with one ^15^N. This diagram summarizes the possible fates of both ^13^C and ^15^N, and for studies using glutamine tracer that only contains one isotope, such as ^15^N, but not ^13^C, the M+n would only indicate the incorporation of n ^15^N. The green line indicates oxidative TCA and related reactions, while the pink line indicates the reductive TCA and related reactions. Dashed lines indicate multi-step reactions.

M+n denotes the incorporation of n ^13^C. For simplicity, this schematic does not take into consideration complicated situations, such as tracer recycling or multiple rounds of the TCA cycle. Furthermore, this diagram can serve as a reference when downstream metabolites are utilized as tracers. For instance, when acetyl-CoA is labeled using [U-13C]glucose, a similar labeling pattern of acetylated proteins or lipids is expected as when acetyl-CoA is labeled using [^13^C_2_]acetate. Dashed lines indicate multi-step reactions.

Fatty acid tracers: Due to the metabolic flexibility of many types of cancer, multiple tracers are often employed in the same study to gain a comprehensive understanding of metabolic adaptations. Certain cancer cell populations acquire the ability to utilize alternative nutrients as part of resistance mechanisms to chemotherapy. For example, resistance to cisplatin, a platinum-based chemotherapy drug, has been associated with increased fatty acid accumulation and beta-oxidation for energy production [154]. Fatty acid uptake and glucose anabolism were monitored using deuterium tracers ([D_31_]-palmitic acid, [D_34_]-oleic acid, or [D_7_]-glucose) through hyperspectral stimulated Raman scattering imaging of C-D bonds in cisplatin-resistant and cisplatin-sensitive cells [154]. The results revealed a higher uptake of palmitic and oleic acid fatty acids and a decreased uptake of glucose in resistant cells. These findings underscore the reliance on fatty acid accumulation for energy production in resistant cells, thus presenting novel targets for drug development [154]. 

Nucleoside tracers: PDAC is highly lethal due to resistance to many chemotherapy treatments [155]. In one study, PDAC is characterized by elevated levels of uridine phosphorylase 1 (UPP1), an enzyme that cleaves uridine to ribose-1-phosphate and uracil. In vivo and in vitro isotope tracing using [^13^C_5_]uridine with uniformly labeled ribose carbon demonstrated labeling of ATP (M+5), AMP (M+5), ADP (M+5), and NAD^+^ (M+5, M+10), confirming the utilization of uridine metabolism for the ribosylation of adenine. Additionally, ^13^C labeling of glycolytic, PPP, and TCA cycle intermediates was observed, indicating that uridine can serve as an alternative energy and carbon source [156]. 

### 2.3. Uncovering Mechanisms of Resistance to Cancer Treatments through Stable Isotope Tracing Analysis

Challenges in the development of cancer therapies: The development of cancer therapeutics is challenging, partly due to the high resistance of cancer cells to treatments and their metabolic plasticity [9,10,157,158,159,160,161,162]. To address this, researchers have turned to comparing metabolic profiles using isotope tracing between resistant and sensitive cancer cells. By employing isotope tracers, researchers can track the fate of labeled metabolites within cancer cells, revealing metabolic adaptations that contribute to treatment resistance. Another critical consideration in drug development is adverse effects either due to off-target activity or unintended actions in normal tissues [9,163,164]. As a result, there is a growing trend toward using multiple inhibitors targeting different metabolic enzymes at tolerable doses to achieve minimal adverse effects and enhance antitumor efficacy [165,166]. Insights gained from isotope tracing studies can inform the development of combination therapies that target both the genetic and metabolic vulnerabilities of cancer cells, thereby enhancing treatment efficacy and overcoming resistance. In this section, we explore studies employing stable isotope tracing to uncover resistance mechanisms in cancer cells, with a particular emphasis on the utilization of ^13^C- or ^15^N-labeled glutamine, since glucose tracing has been extensively studied.

Resistant mechanisms of GLS inhibitors: Despite being recognized as a potent and well-tolerated GLS inhibitor, CB-839 has shown mixed results in clinical trials [167,168]. While it has demonstrated efficacy in certain patients [167], a randomized clinical trial investigating its combination with cabozantinib in metastatic renal cell cancer (RCC) did not improve efficacy [168]. Similarly, during a phase II clinical trial involving patients with stage IV non-small cell lung cancer (NSCLC) tumors carrying loss-of-function mutations in KEAP1 (KEAPSAKE trial, NCT04265534), CB-839 did not show advantageous effects compared to standard-of-care immunotherapy. To understand the resistant mechanisms, [U-^13^C_5_]glutamine tracing was performed in mice carrying liver tumors with the deletion of GLS1 and a reduced expression of GLS2 (Gls1KO/shGls2). The presence of a substantial fraction of the M+4 malate derived from [U-^13^C_5_]glutamine suggests that other enzymes can utilize glutamine as an amide nitrogen donor and generate glutamate [121]. This finding led to the approach of synergistic inhibition of both glutaminases and compensatory amidotransferases to block glutamine catabolism and the proliferation of cancer cells in vitro and in vivo [121]. In another study, infusions of [5-^15^N]glutamine in clear cell RCC tumorgrafts unveiled sustained amidotransferase activity despite glutaminase inhibition by CB-839 [118]. When JHU-083, a prodrug of DON, was tested, [U-^13^C_5_, ^15^N_2_]glutamine tracing demonstrated a greater inhibition of the ^15^N labeling of metabolites catalyzed by amidotranferases than the ^13^C labeling of TCA cycle intermediates, suggesting that JHU-083 inhibited amidotransferases more effectively than GLS, subsequently resulting in more robust inhibition of tumor growth in both immunocompromised and immunocompetent mice than CB-839 [118].

Identifications of resistant cells to chemotherapy and radiotherapy: Glutamine tracing experiments were also performed to understand the resistant mechanisms of other types of cancer treatments. For example, [U-^13^C_5_]glutamine or [^15^N_2_]glutamine was injected intravenously into acute myeloid leukemia (AML)-bearing mice with vehicle or induction chemotherapy (iCT) for assessing labeled metabolites in AML cells or residual AML cells surviving iCT treatment [169]. The results revealed an increase in glutamine metabolism and ^15^N incorporation into pyrimidines in residual AML compared to the control AML cells [169]. The subsequent inhibition of de novo pyrimidine synthesis by brequinar (BRQ) post-iCT treatment significantly prolonged the survival of AML-bearing mice compared to iCT treatment alone, suggesting that glutamine metabolism drives AML chemoresistance by supporting pyrimidine synthesis [169]. Metabolomics and [U-^13^C_5_]glutamine tracing analysis of ovarian cancer cells, which were sensitive or resistant to cisplatin treatment, revealed that glutamine metabolism is enhanced in cisplatin-resistant ovarian cancer cells [170]. The inhibition of glutaminase by compound 968 partially reversed the resistance to cisplatin treatment in these ovarian cancer cells [170]. In another study, metabolomics and a [U-^13^C_5_]glutamine tracing analysis of prostate cancer cells sensitive or resistant to radiotherapy demonstrated that radioresistant prostate cancer cells and prostate cancer stem cells have a high glutamine demand, and reducing glutamine metabolism via glutamine depletion or the inhibition of critical regulators of glutamine utilization restored prostate cancer cell radiosensitization [171].

Mechanisms of resistance to immunotherapy: It is increasingly recognized that nutrient availability and metabolic activities in the tumor microenvironment play a role in determining immune cell function and antitumor immunity [145,172,173,174]. [U-^13^C_5_]glutamine was used to monitor the glutamine uptake by dendritic cells, regulated by the transporter SLC38A2 [173]. Limited glutamine availability in the tumor microenvironment or impaired glutamine uptake due to SLC38A2 deletion compromised antitumor immunity [173]. In a separate study, inhibiting glutamine utilization by tumor cells enhanced antitumor T cell responses, and the combination of a GLS inhibitor, CB-839, with a glutamine antagonist, DRP-104, demonstrated a synergistic effect, enhancing the response to anti-PD1 checkpoint inhibitor therapy in Keap1 mutant lung tumors [145]. The [U-^13^C_5_]glutamine tracing analysis suggests distinct actions of CB-839 and DRP-104 in vivo and specifically, that DRP-104 impairs tumor proliferation by inhibiting glutamine-dependent nucleotide synthesis rather than inhibiting glutaminolysis [145].

Identifications of resistant cells to hormone therapy: The contribution of metabolic alterations to hormonal therapy resistance in prostate cancer remains poorly understood. A [U-^13^C_5_]glutamine tracing analysis played an important role in revealing the types of prostate cancer cells in which glutamine metabolism was more susceptible to CB-839 and pinpointing the isoform of GLS1 that was more sensitive to CB-839 [140]. These experiments also led to the discovery that GLS1 isoform switch is one of the key mechanisms in hormonal therapy resistance and disease progression [140]. 

Mechanisms of resistance to targeted therapy: Epidermal growth factor receptor tyrosine kinase inhibitors (EGFR-TKIs), which target early-stage lung cancer with EGFR mutations, have been among the most successful targeted cancer therapies [175,176,177]. However, the development of resistance to EGFR-TKIs poses a significant challenge in clinical management [178]. A [U-^13^C_5_]glutamine tracing analysis of acquired EGFR-TKI-resistant lung cancer cell lines has unveiled a critical dependency on glutamine for supporting TCA cycle activity and glutathione (GSH) biosynthesis [179]. This finding offers valuable insights into the metabolic adaptations underlying EGFR-TKI resistance [179].

### 2.4. Current Clinical Trials Employing Stable Isotope Tracing Techniques

Alongside stable isotope tracing investigations into glucose and glutamine metabolism in vitro and mouse models of cancer, patient clinical trials are progressing to monitor anticancer drug actions, including target engagement and off-target effects, as well as metabolic adaptations within the human body [1]. Table 2 summarizes current clinical trials utilizing labeled glucose and glutamine, outlining the cancer type, isotope tracer used, and the measurement technique for metabolite analysis. Stable isotope tracing analysis emerges as a catalyst for discovery and therapy in the clinical setting [180]. The administration of labeled glucose or glutamine intravenously to cancer patients before surgery or tumor biopsy provides insights into cancer cell reliance on these molecules for proliferation and survival across various cancer types. For example, in an ongoing clinical trial (NCT05296421) sponsored by the State University of New Jersey to identify targetable metabolic pathways that sustain pancreatic cancer, patients will receive [U-^13^C_6_]glucose intravenously until the time of biopsy, followed by metabolite extraction and analysis using LC-MS. In a separate study [181], to understand the association of glutamine anaplerosis with the progression of pre-malignant to malignant plasma cells, tracing with [U-^13^C_5_]glutamine was performed and revealed that the flux of glutamine into the TCA cycle was elevated in malignant bone marrow plasma cells compared to their pre-malignant counterparts relative to the remaining paired bone marrow mononuclear cells. This finding aligns with RNA sequencing results indicating higher mRNA expression levels of glutamine transporters, such as ASCT2 and SN2, in malignant bone marrow plasma cells compared to pre-malignant ones [181].

## 3. Limitations and Future Directions

While stable isotope tracing analysis is a valuable tool in metabolic research, it does have certain limitations:

Technical limitations, such as limited spatial resolution: Stable isotope tracing techniques typically provide bulk measurements of metabolic fluxes within tissues or whole organisms. They may lack the spatial resolution required to study metabolic dynamics at the cellular or subcellular level. Metabolic pathways are compartmentalized within cells and tissues, and tracer kinetics may differ between compartments. Failure to account for compartmentalization can lead to inaccuracies in flux estimates and the misinterpretation of metabolic fluxes. The stable isotope-labeling of essential nutrients in cell culture subcellular fractionation (SILEC-SF) and immunopurification-based subcellular fraction approaches show promise in quantifying metabolites in subcellular compartments [182,183]. Additionally, considerable progress has been achieved in the field of single-cell metabolomics [59,184,185,186,187]. 

Perturbations of systemic and tumor metabolism through the administration of a stable isotope tracer in vivo: The methods of fasting, administration of tracers, tracer dosage, and other experimental parameters can all exert significant influences on both systemic and tumor metabolism, potentially leading to variability in experimental outcomes [188]. It is crucial to optimize tracing protocols tailored to the specific metabolic pathway under investigation. 

Complexity of interpretation: Factors such as tracer recycling and interorgan conversion of the tracer increase the complexity of metabolic networks in vivo [188,189,190,191], posing challenges to the accurate interpretation of tracer kinetics and fluxes. Furthermore, the rapid exchange of labeled atoms can occur without the net conversion of substrates to products when there is significant reaction reversibility. A comprehensive interpretation of stable isotope tracing data requires sophisticated mathematical modeling and analytical techniques [99,189,191,192,193]. 

Assumptions and limitations of tracer incorporation: Stable isotope tracer studies often assume rapid mixing and equilibration of the tracer throughout the target tissue, which may not always hold true. In addition, many studies assume that the labeling of downstream products has reached a steady state without direct confirmation, which can dramatically impact the assumptions and modeling approaches used to interpret the results. Tracer incorporation rates can vary depending on factors such as tissue type, metabolic state, and the specific pathway being investigated [22]. Hence, pilot studies are necessary to determine the metabolic dynamics and ensure a properly optimized tracing protocol. 

Cost and difficulties in clinical translation: Stable isotope tracing experiments can be expensive and require specialized equipment and technical expertise for tracer administration, sample collection, isotope analysis, and data interpretation. These practical considerations are amplified in the clinical setting in which large doses of pharmaceutical-grade tracers are required and invasive or burdensome protocols are unlikely to be tolerated by patients. This could hinder access for researchers lacking adequate resources and training. However, advancements in developing more sensitive methods have the potential to reduce costs. Furthermore, collaborative efforts among different teams with shared or complementary interests could help distribute the financial burden.

## 4. Conclusions

In summary, stable isotope tracing analysis has emerged as a widely utilized tool for mechanistic studies aimed at understanding various facets of cancer metabolism. This includes exploring cancer metabolic rewiring, metabolic heterogeneity, the interplay with the immune system in the tumor microenvironment, adaptive responses to cancer therapies, and more. Despite its current limitations, including the technical challenges and the high cost, stable isotope tracing remains a powerful methodology for investigating metabolic pathways and dynamics in both health and disease contexts. Integrating tracer data with other omics and imaging approaches holds promise in mitigating some of these limitations and offering a more comprehensive understanding of metabolic regulation. Furthermore, the ongoing advancements in clinical applications not only enhance but also extend the significance of stable isotope tracing beyond experimental models in cancer research.

## Figures and Tables

**Table 2:** Current clinical trials using isotope tracing analysis.

Cancer Type	Isotope Tracer	Analytical Technique	ClinicalTrials.gov Identifier
Pancreatic Ductal Adenocarcinoma	[U-^13^C_6_]glucose	LC-MS	NCT05296421
Multiple Myeloma	[U-^13^C_5_]glutamine	LC-MS	NCT03119883 [181]
Hormone Receptor Positive (HR+)/Her 2 Negative (Her2-) Breast Cancer	[U-^13^C_6_]glucose	LC-MS	NCT05736367
Kidney or Urothelial Cancer	[U-^13^C_6_]glucose, [U-^13^C_2_]acetate, [U-^13^C_3_]lactate, [U-^13^C_5_]glutamine, [U-^13^C_6_]fructose	Unspecified	NCT04623502
Brain Cancer	[U-^13^C_6_]glucose, [1,2-^13^C_2_]glucose	^13^C NMR	NCT01668082

## Data Availability

No new data were created or analyzed in this study. Data sharing is not applicable to this article.
